# Lobular Difference in Heritability of Brain Atrophy among Elderly Japanese: A Twin Study

**DOI:** 10.3390/medicina58091250

**Published:** 2022-09-09

**Authors:** Soichiro Saeki, Helga Szabo, Rie Tomizawa, Adam D. Tarnoki, David L. Tarnoki, Yoshiyuki Watanabe, Osaka Twin Research Group, Chika Honda

**Affiliations:** 1 Center Hospital of the National Center for Global Health and Medicine, Tokyo 162-8655, Japan; 2 Department of Global and Innovative Medicine, Graduate School of Medicine, Osaka University, Osaka 565-0871, Japan; 3 Medical Imaging Centre, Semmelweis University, 1082 Budapest, Hungary; 4 Center for Twin Research, Graduate School of Medicine, Osaka University, Osaka 565-0871, Japan; 5 School of Nursing, Graduate School of Nursing, Osaka Metropolitan University, Osaka 545-8585, Japan; 6 Department of Radiology, Shiga University of Medical Science, Shiga 520-2192, Japan; 7 Department of Public Health Nursing, Shiga University of Medical Science, Shiga 520-2192, Japan

**Keywords:** brain volume, brain atrophy, twin study, dementia, Japan

## Abstract

*Background and Objectives*: Brain atrophy is related to cognitive decline. However, the heritability of brain atrophy has not been fully investigated in the Eastern Asian population. *Materials and Methods*: Brain imaging of 74 Japanese twins registered in the Osaka University Twin Registry was conducted with voxel-based morphometry SPM12 and was processed by individual voxel-based morphometry adjusting covariates (iVAC) toolbox. The atrophy of the measured lobes was obtained by comparing the focal volume to the average of healthy subjects. Classical twin analysis was used to measure the heritability of its z-scores. *Results*: The heritability of brain atrophy ranged from 0.23 to 0.97, depending upon the lobes. When adjusted to age, high heritability was reported in the frontal, frontal-temporal, and parietal lobes, but the heritability in other lobes was lower than 0.70. *Conclusions*: This study revealed a relatively lower heritability in brain atrophy compared to other ethnicities. This result suggests a significant environmental impact on the susceptibility of brain atrophy the Japanese. Therefore, environmental factors may have more influence on the Japanese than in other populations.

## 1. Introduction

Brain volume changes over the course of life [1,2]. Especially for the elderly, brain volume is known to decrease, which is known to be associated with cognitive decline [3] and memory loss [4]. As the Japanese society has been the frontrunner of aging in the world [5], such age-related issues of Japan will be of great interest for the entire global population.

Previous studies have identified that brain volume is highly heritable [2,6,7,8,9], but the range in its heritability differs between the area of the brain. However, such investigations have not been widely conducted in the Eastern Asian population. Lukies et al. [10] previously reported heritability of brain volume from Japan, but this study only provided the heritability of total brain volume and several regions of the brain, including the gray matter in white matter.

Although there are several methods in estimating heritability [11], twin studies are ideal as they can adjust for the confounding of environmental factors [12,13]. Thus, we utilized the classical twin study to identify the differences in heritability of brain atrophy compared to normal control subjects within each lobe of the brain.

## 2. Materials and Methods

### 2.1. Subjects

74 Japanese twins (37 pairs), comprised of 20 monozygotic (MZ) twin pairs and 17 dizygotic (DZ) twin pairs over 40 years old at the time of measurement were recruited from the Osaka University Twin Registry established by the Center for Twin Research, Graduate School of Medicine, Osaka University [14]. As the data used for this study was already obtained for another study [10], subjects were provided with the opportunity to opt-out from the study by mail. We assessed 20 pairs of MZ twins (mean age ± SD = 61.10 ± 8.74 years) and 16 pairs of DZ twins (mean age ± SD = 63.19 ± 13.16 years). The MZ group was made up of nine male and 19 female twin pairs, while the DZ group was made up of six male and six female twin pairs. Because one of the 17 DZ pairs contained a male-female twin, this pair was excluded from the classical twin analysis, which was carried out for 72 twins (36 pairs: 20 MZ twins and 16 DZ twins). MZ and DZ were both 50 percent male and 50 percent female. Age, gender, and zygosity were all reconfirmed in the dataset for this study. This study was approved by the Ethical committee of the Graduate School of Medicine, Osaka University (approval number 21143).

### 2.2. Brain Imaging

Brain imaging was conducted with voxel-based morphometry SPM12 (Wellcome Department of Cognitive Neurology, London) and was processed by the individual voxel-based morphometry adjusting covariates (iVAC) toolbox [15]. 3D T1-weighted images with inversion recovery gradient echo sequence using 3.0T MRI unit were obtained at the 1 mm^3^ iso-voxel resolution. Z-scores were produced and mapped, to represent the difference, in standard deviations, of focal volumes compared to averages from 232 healthy subjects, adjusted for age and sex. In addition, analysis was conducted comparing each of the examinee’s lobular differences to the other.

### 2.3. Statistical Analysis

We presented each continuous variable with its mean and standard deviation (SD), and each categorical variable is represented with numbers and percentages. T-test was applied to the continuous variables, while the chi-square test was applied to the categorical variables.

The correlation of each regional z-score between twin pairs was evaluated. Univariate twin analysis was performed using the Mets package [16] on R. Interclass correlations for MZ (rMZ) and DZ (rDZ) were calculated. Equation modeling was constructed to classify the variance into additive genetic effects (A), dominance genetic effects, shared environmental effects (C), and unique environmental effects (E). This model is based upon the assumption that MZ pairs share nearly 100% of their genome and the DZ pairs on average share 50% of their genomes. This model also requires all genetic variance to be additive, and the environmental covariance and total variances of MZ and DZ are equal [17]. The best fit model based on the Akaike Information Criteria was selected as the reporting model, analyzed with adjustment for age.

Statistical analysis was performed using EZR [18] (Saitama Medical Center, Jichi Medical University, Saitama, Japan), a graphical user interface for R [19]. The R version used was 3.6.1. The *p*-value < 0.05 was determined as statistically significant.

## 3. Results

Table 1 describes the comparison of the continuous and categorical variables between the zygosity of twins.

Results of the univariate twin analysis are shown in Table 2. Intra-twin correlations show that models other than the CE model show a stronger correlation between the MZ than the DZ.

The best fit model for the group with all subjects was either the AE, CE, or the ACE model, depending on the analyzed lobe (Table 2a). Compared to the original model, the adjusted model compared to a 30-year-old model reported higher heritability in most of the lobes (Table 2b). The heritability of brain atrophy ranged from 0.23 to 0.97, depending upon the lobes. When adjusted for age, high heritability was reported in the frontal, frontal-temporal, and parietal lobes, but the heritability in other lobes was lower than 0.70.

## 4. Discussion

Our study reported that brain atrophy is heritable in many lobes, with z-scores adjusted for age and sex, compared to the 30-year-old brain model. More precisely, the heritability of brain atrophy ranged from 0.23 to 0.97, depending upon the lobes. When adjusted to age, high heritability was reported in the frontal, frontal-temporal, and parietal lobes, but the heritability in other lobes were lower than 0.70, as observed from the homogeneity of the variances between the MZ and DZ. To the best of the authors’ knowledge, no other twin studies have been conducted to evaluate focal brain atrophy in comparison to normal control subjects.

The fact that environmental factors play a larger role in determining the degree of atrophy in many parts of the brain lobes suggests that further interventions may be feasible in preventing such volume loss. In our model, differences in the extent of environmental influences between the lobes were observed. Namely, the cerebellum, temporal, limbic, and occipital lobe had significant environmental factors while other lobes were influenced by genetic factors. Previous studies have demonstrated several potential environmental factors that may influence the volumes of these regions of the brain. For example, atrophy of the cerebellum is estimated to be associated with the exposure to substances such as pesticides and nicotine [20]. Body mass index is also known to be associated with the progression of atrophy of the medial temporal lobe in patients at risk of Alzheimer’s disease [21]. The volume of the hippocampus, a component of the limbic system, is reported to be influenced by stress and stress coping strategies [22]. Volume of both the limbic and occipital lobes are known to decrease in diseases such as argyrophilic grain disease [23]. These regions of the brain may be more susceptible to environmental influences. Such environmental factors, in addition to other factors, as well as its degree of the influence should be investigated in future studies.

Compared to previous reviews and studies by other ethnicities [1,6,9], our results of the Japanese population report a higher environmental contribution to brain volume loss, although a direct comparison is difficult, as the age of the sample is different among each of the studies. For example, high blood pressure during adulthood correlates with brain volume loss according to a British cohort study [24], and such environmental factors should be investigated among the Japanese population as well. Although further investigation would be necessary to reach a more concrete understanding, our findings should be regarded as another suggestion of evidence referring to differences between race and ethnicity in the changes humans experience during the course of aging.

This study has several limitations. Firstly, the nature of the study limits the participants to a relatively healthy population, as the participants must be able to come to the recruitment sites for the study. The relatively ill population may have not been able to participate in our study. Secondly, the adjusted model was created from the brain volume of a rather young generation, on the assumption that brain volume decreases according to a linear model. Thirdly, the sample size of the study remains relatively small, which may have been a reason for reporting a wide-ranged 95% CI. This has limited our study from investigating associations within subgroups such as sex, different atrophy forms and other genetic and environmental factors. Additionally, this may have led to the difference in the mean of the z-score between the MZ and DZ. Furthermore, this may have led to an underrepresentation of non-additive genetic influences. Fourthly, this study is limited to the Japanese population.

## 5. Conclusions

In conclusion, our study revealed a relatively lower heritability in brain atrophy compared to other ethnicities. This result suggests a significant environmental impact on the susceptibility of brain atrophy the Japanese. Therefore, environmental factors may have more influence on the Japanese than in other populations. Further studies of such environmental impacts and interventions should be conducted for the prevention of dynamic changes of brain atrophy among elderly people in Japan.

## Figures and Tables

**Table 1:** Comparison of variables between types of twin zygosity according to age.

			Original			Adjusted by Age		
Zygosity			MZ	DZ	*p*-Value	MZ	DZ	*p*-Value
*n*			40	32		40	32	
Gender [*n*]		male	20	16	1	20	16	1
Age [years] (SD)			61.10 (8.74)	63.19 (13.16)	0.423	61.10 (8.74)	63.19 (13.16)	0.423
Brain Volume [z-score] (95% CI)	Cerebellum Posterior Lobe	left	−0.20 [−1.90, 0.62]	−0.87 [−1.67, −0.14]	<0.001	1.15 [−0.89, 2.65]	0.81 [−0.58, 1.92]	0.063
	Temporal Lobe		−0.12 [−1.20, 0.81]	−0.55 [−1.15, 0.20]	<0.001	1.00 [−0.40, 2.25]	0.72 [−0.80, 2.03]	0.065
	Limbic Lobe		−0.21 [−1.12, 1.26]	−1.21 [−2.23, 0.13]	<0.001	1.28 [−0.18, 2.52]	0.19 [−1.40, 3.21]	<0.001
	Frontal Lobe		−0.04 [−1.10, 0.71]	−0.77 [−1.68, 0.44]	<0.001	1.21 [−0.01, 3.32]	0.38 [−1.01, 2.56]	<0.001
	Sub-lobar		0.04 [−1.43, 0.96]	−1.21 [−2.24, 0.17]	<0.001	1.20 [−0.31, 3.09]	0.22 [−1.53, 3.47]	0.003
	Cerebellum Anterior Lobe		−0.07 [−1.14, 1.13]	−0.93 [−2.09, 0.31]	<0.001	1.40 [0.05, 2.44]	0.34 [−1.22, 2.04]	<0.001
	Occipital Lobe		−0.10 [−1.02, 0.83]	−1.10 [−1.98, 0.36]	<0.001	0.97 [−0.31, 2.42]	0.13 [−1.29, 1.91]	<0.001
	Frontal-Temporal Space		−0.13 [−1.19, 1.11]	−0.56 [−2.23, 0.92]	0.015	1.87 [−0.40, 4.79]	0.77 [−0.98, 2.93]	<0.001
	Parietal Lobe		−0.06 [−1.06, 0.55]	−0.74 [−1.42, 0.59]	<0.001	0.87 [0.17, 2.61]	0.19 [−1.07, 2.32]	<0.001
	Cerebellum Posterior Lobe	right	−0.19 [−1.70, 0.86]	−0.57 [−1.67, 0.09]	0.001	0.81 [−0.58, 1.92]	0.81 [−0.58, 1.92]	0.044
	Temporal Lobe		−0.20 [−1.05, 0.49]	−0.57 [−1.34, 0.25]	<0.001	0.72 [−0.80, 2.03]	0.72 [−0.80, 2.03]	0.1
	Limbic Lobe		−0.08 [−1.17, 0.89]	−1.15 [−2.06, 0.06]	<0.001	0.19 [−1.40, 3.21]	0.19 [−1.40, 3.21]	<0.001
	Frontal Lobe		0.03 [−0.97, 0.75]	−0.87 [−1.71, 0.44]	<0.001	0.38 [−1.01, 2.56]	0.38 [−1.01, 2.56]	<0.001
	Sub-lobar		−0.10 [−1.23, 0.68]	−1.30 [−2.31, 0.37]	<0.001	0.22 [−1.53, 3.47]	0.22 [−1.53, 3.47]	0.002
	Cerebellum Anterior Lobe		0.01 [−1.44, 0.80]	−0.80 [−2.09, 0.19]	<0.001	0.34 [−1.22, 2.04]	0.34 [−1.22, 2.04]	<0.001
	Occipital Lobe		0.02 [−0.97, 1.28]	−1.05 [−1.58, −0.07]	<0.001	0.13 [−1.29, 1.91]	0.13 [−1.29, 1.91]	<0.001
	Frontal-Temporal Space		−0.23 [−2.03, 1.50]	−1.01 [−2.49, 0.53]	0.001	0.77 [−0.98, 2.93]	0.77 [−0.98, 2.93]	0.017
	Parietal Lobe		−0.16 [−0.87, 0.75]	−0.76 [−1.58, 0.93]	<0.001	0.19 [−1.07, 2.32]	0.19 [−1.07, 2.32]	<0.001

Brain volume values represent the z-score of lobular volumes adjusted for age and gender. 95% CIs are indicated in parentheses. Legends: SD standard deviation; CI confidence interval.

**Table:** (**a**) Univariate ADCE model fitting of brain atrophy of the original model.

			Original					
n			Model	rMZ	rDZ	A	C	E
Brain Atrophy [z-score] (95% CI)	Cerebellum Posterior Lobe	left	CE	0.54 [0.17, 0.78]	0.42 [−0.04, 0.74]	-	0.68 [0.50, 0.85]	0.32 [0.15, 0.50]
	Temporal Lobe		AE	0.81 [0.59, 0.92]	0.48 [0.03, 0.77]	0.75 [0.59, 0.92]	-	0.25 [0.08, 0.41]
	Limbic Lobe		AE	0.73 [0.45, 0.88]	0.17 [−0.31, 0.58]	0.88 [0.80, 0.96]	-	0.12 [0.04, 0.20]
	Frontal Lobe		AE	0.73 [0.44, 0.87]	−0.09 [−0.53, 0.38]	0.86 [0.76, 0.96]	-	0.14 [0.04, 0.24]
	Sub-lobar		AE	0.70 [0.41, 0.86]	0.46 [0.00, 0.75]	0.88 [0.80, 0.96]	-	0.12 [0.04, 0.20]
	Cerebellum Anterior Lobe		CE	0.68 [0.38, 0.86]	0.64 [0.26, 0.85]	-	0.845 [0.75, 0.94]	0.16 [0.06, 0.25]
	Occipital Lobe		CE	0.53 [0.15, 0.77]	0.43 [−0.03, 0.74]	-	0.80 [0.68, 0.92]	0.20 [0.08, 0.32]
	Frontal-Temporal Space		AE	0.46 [0.00, 0.76]	0.32 [−0.07, 0.63]	0.49 [0.17, 0.82]	-	0.51 [0.18, 0.83]
	Parietal Lobe		AE	0.88 [0.74, 0.95]	0.09 [−0.38, 0.52]	0.95 [0.91, 0.99]	-	0.05 [0.01, 0.09]
	Cerebellum Posterior Lobe	right	AE	0.61 [0.31, 0.80]	0.40 [−0.09, 0.74]	0.62 [0.39, 0.85]	-	0.38 [0.15, 0.61]
	Temporal Lobe		AE	0.75 [0.48, 0.89]	0.47 [0.03, 0.76]	0.71 [0.51, 0.91]	-	0.29 [0.09, 0.49]
	Limbic Lobe		ACE	0.62 [0.28, 0.82]	0.29 [−0.19, 0.66]	0.35 [−0.16, 0.86]	0.48 [−0.01, 0.97]	0.16 [0.04, 0.28]
	Frontal Lobe		AE	0.71 [0.42, 0.87]	−0.14 [−0.56, 0.33]	0.87 [0.78, 0.96]	-	0.13 [0.04, 0.22]
	Sub-lobar		AE	0.76 [0.50, 0.89]	0.37 [−0.10, 0.70]	0.91 [0.85, 0.97]	-	0.09 [0.03, 0.15]
	Cerebellum Anterior Lobe		CE	0.64522 [0.32, 0.84]	0.73 [0.41, 0.89]	-	0.79 [0.67, 0.91]	0.21 [0.09, 0.33]
	Occipital Lobe		CE	0.79 [0.55, 0.91]	0.61 [0.22, 0.84]	-	0.85 [0.75, 0.94]	0.15 [0.06, 0.25]
	Frontal-Temporal Space		AE	0.40 [−0.02, 0.70]	0.31 [0.17, 0.67]	0.58 [0.31, 0.85]	-	0.42 [0.15, 0.69]
	Parietal Lobe		AE	0.74 [0.48, 0.88]	−0.01 [−0.46, 0.45]	0.90 [0.82, 0.97]	-	0.10 [0.03, 0.18]

95% CIs are indicated in parentheses. Legends: rMZ intrapair correlation in monozygotic twins; rDZ intrapair correlation in dizygotic twins; A additive genetic factors; D dominant genetic factors; C common environmental factors; E unique environmental factors; AIC Akaike Index Criterion; SD standard deviation; CI confidence interval.

**Table:** (**b**) Univariate ADCE model fitting of brain atrophy of the adjusted model.

			Adjusted					
n			Model	rMZ	rDZ	A	C	E
Brain Atrophy [z-score] (95% CI)	Cerebellum Posterior Lobe	left	CE	0.56 [0.19, 0.79]	0.59 [0.19, 0.82]	-	0.70 [0.53, 0.87]	0.30 [0.13, 0.47]
	Temporal Lobe		ACE	0.70 [0.41, 0.86]	0.76 [0.47, 0.90]	0.34 [−0.04, 0.72]	0.55 [0.18, 0.93]	0.10 [0.03, 0.18]
	Limbic Lobe		ACE	0.75 [0.48, 0.89]	0.83 [0.59, 0.93]	0.27 [−0.03, 0.57]	0.65 [0.36, 0.95]	0.08 [0.02, 0.13]
	Frontal Lobe		AE	0.77 [0.53, 0.90]	0.66 [0.30, 0.86]	0.93 [0.87, 0.98]	-	0.07 [0.02, 0.13]
	Sub-lobar		ACE	0.76 [0.50, 0.89]	0.95 [0.86, 0.98]	0.31 [0.01, 0.61]	0.62 [0.32, 0.93]	0.06 [0.01, 0.11]
	Cerebellum Anterior Lobe		CE	0.69 [0.39, 0.86]	0.79 [0.53, 0.92]	-	0.83 [0.73, 0.93]	0.17 [0.07, 0.27]
	Occipital Lobe		CE	0.69 [0.39, 0.86]	0.94 [0.85, 0.98]	-	0.84 [0.75, 0.94]	0.16 [0.06, 0.25]
	Frontal-Temporal Space		AE	0.43 [0.03, 0.72]	0.37 [−0.10, 0.71]	0.77 [0.61, 0.93]	-	0.23 [0.07, 0.39]
	Parietal Lobe		AE	0.92 [0.82, 0.97]	0.83 [0.61, 0.93]	0.97 [0.95, 0.99]	-	0.03 [0.01, 0.05]
	Cerebellum Posterior Lobe	right	CE	0.62 [0.27, 0.82]	0.59 [0.19, 0.82]	-	0.71 [0.56, 0.87]	0.29 [0.13, 0.44]
	Temporal Lobe		ACE	0.70 [0.41, 0.86]	0.76 [0.47, 0.90]	0.30 [−0.08, 0.69]	0.57 [0.20, 0.95]	0.12 [0.03, 0.21]
	Limbic Lobe		ACE	0.66 [0.34, 0.84]	0.83 [0.59, 0.93]	0.23 [−0.07, 0.53]	0.67 [0.38, 0.97]	0.09 [0.02, 0.17]
	Frontal Lobe		AE	0.78 [0.55, 0.90]	0.66 [0.30, 0.86]	0.92 [0.87, 0.98]	-	0.08 [0.02, 0.13]
	Sub-lobar		AE	0.82 [0.61, 0.92]	0.95 [0.86, 0.98]	0.95 [0.91, 0.99]	-	0.05 [0.01, 0.09]
	Cerebellum Anterior Lobe		ACE	0.65 [0.32, 0.84]	0.79 [0.53, 0.92]	0.23 [−0.10, 0.56]	0.66 [0.34, 0.97]	0.11 [0.03, 0.20]
	Occipital Lobe		CE	0.82 [0.61, 0.92]	0.94 [0.85, 0.98]	-	0.87 [0.80, 0.95]	0.13 [0.05, 0.20]
	Frontal-Temporal Space		AE	0.46 [0.06, 0.73]	0.37 [−0.10, 0.71]	0.66 [0.45, 0.88]	-	0.34 [0.12, 0.55]
	Parietal Lobe		AE	0.83 [0.63, 0.93]	0.83 [0.61, 0.93]	0.94 [0.89, 0.98]	-	0.06 [0.02, 0.11]

95% Cis are indicated in parentheses. Legends: rMZ intrapair correlation in monozygotic twins; rDZ intrapair correlation in dizygotic twins; A additive genetic factors; D dominant genetic factors; C common environmental factors; E unique environmental factors; AIC Akaike Index Criterion; SD standard deviation; CI confidence interval.

## Data Availability

The dataset used for the study is available from the corresponding author on reasonable request.
